# Pulse versus nonpulse steroid regimens in patients with coronavirus disease 2019: A systematic review and meta‐analysis

**DOI:** 10.1002/jmv.27824

**Published:** 2022-05-09

**Authors:** Waleed Khokher, Azizullah Beran, Saffa Iftikhar, Saif‐Eddin Malhas, Omar Srour, Mohammed Mhanna, Sapan Bhuta, Dipen Patel, Nithin Kesireddy, Cameron Burmeister, Elizabeth Borchers, Ragheb Assaly, Fadi Safi

**Affiliations:** ^1^ Department of Internal Medicine University of Toledo Toledo Ohio USA; ^2^ Department of Medicine University of Toledo College of Medicine and Life Sciences Toledo Ohio USA; ^3^ Department of Pulmonary and Critical Care Medicine University of Toledo Toledo Ohio USA

**Keywords:** corticosteroids, COVID‐19, hospital stay, intubation, mortality, pulse dose steroids

## Abstract

Systemic steroids are associated with reduced mortality in hypoxic patients with coronavirus disease 2019 (COVID‐19). However, there is no consensus on the doses of steroid therapy in these patients. Several studies showed that pulse dose steroids (PDS) could reduce the progression of COVID‐19 pneumonia. However, data regarding the role of PDS in COVID‐19 is still unclear. Therefore, we performed this meta‐analysis to evaluate the role of PDS in COVID‐19 patients compared to nonpulse steroids (NPDS). Comprehensive literature search of PubMed, Embase, Cochrane Library, and Web of Science databases from inception through February 10, 2022 was performed for all published studies comparing PDS to NPDS therapy to manage hypoxic patients with COVID‐19. Primary outcome was mortality. Secondary outcomes were the need for endotracheal intubation, hospital length of stay (LOS), and adverse events in the form of superimposed infections. A total of 10 observational studies involving 3065 patients (1289 patients received PDS and 1776 received NPDS) were included. The mortality rate was similar between PDS and NPDS groups (risk ratio [RR]: 1.23, 95% confidence interval [CI]: 0.92–1.65, *p* = 0.16). There were no differences in the need for endotracheal intubation (RR: 0.71, 95%: CI 0.37–1.137, *p* = 0.31), LOS (mean difference: 1.93 days; 95% CI: −1.46–5.33; *p* = 0.26), or adverse events (RR: 0.93, 95% CI: 0.56–1.57, *p* = 0.80) between the two groups. Compared to NPDS, PDS was associated with similar mortality rates, need for endotracheal intubation, LOS, and adverse events. Given the observational nature of the included studies, randomized controlled trials are warranted to validate our findings.

## 1 INTRODUCTION

Coronavirus disease 2019 (COVID‐19), caused by severe acute respiratory syndrome coronavirus 2 (SARS‐CoV‐2) infection, first discovered in China in December 2019, and has become a worldwide pandemic leading to significant morbidity and mortality.^1^, ^2^ The acute respiratory distress syndrome (ARDS) due to viral pneumonitis is one of the leading causes of mortality among patients with COVID‐19.^3^ ARDS occurs in 33% of hospitalized patients with COVID‐19 and 75% of those who required ICU admission.^4^ The average mortality rate among COVID‐19 patients with ARDS is 39% (ranging from 13% to 73%).^3^

In the absence of specific antiviral therapy for COVID‐19, research on the effectiveness of various re‐purposed drugs in COVID‐19 has become an urgent task of scientists and physicians. Exaggerated inflammatory response with cytokine storm is the hallmark of moderate to severe cases of COVID‐19.^5^ In July 2020, the RECOVERY trial showed that low‐dose dexamethasone reduced mortality in patients with COVID‐19 who need oxygen supplementation.^6^ Since then, many studies have been conducted and demonstrated that systemic steroids were associated with reduced mortality in hypoxic patients with COVID‐19.^7^, ^8^ Systemic steroids work by decreasing the expression of pro‐inflammatory cytokines, thus reducing the IL‐6 mediated cytokine storm, which prevents further progression of ARDS.^8^ However, the role of pulse dose steroids (PDS) in ARDS patients is not well established. PDS entails the use of glucocorticoids, usually methylprednisolone (MTP), delivered at very high doses of 10–20 mg/kg or >250 mg/day and as high as 1 g/day.^9^ The use of PDS has been was studied during the SARS and MERS epidemics, with the results being controversial.^10^, ^11^ The theory behind using PDS steroids is that the high dose of steroids can counter the hyperinflammatory phase COVID‐19 and can help to reduce mortality.^12^, ^13^

Although systemic steroids have shown a mortality benefit in COVID‐19, there is no consensus on the doses of steroids therapy in these patients. Newer studies showed that PDS could reduce the progression of COVID‐19.^14^, ^15^, ^16^ Several studies have compared PDS versus nonpulse dose steroids (NPDS) with conflicting results.^14^, ^17^, ^18^, ^19^ Data regarding the role of PDS in COVID‐19 are still unclear. Therefore, we performed this meta‐analysis to evaluate the effect of PDS versus NPDS on the clinical outcomes of patients with COVID‐19 pneumonia.

## 2 METHODS

### 2.1 Data sources and search strategy

We performed a comprehensive search for published studies indexed in PubMed/MEDLINE, EMBASE, Cochrane Central Register of Controlled Trials, and Web of Science from inception to February 10, 2022. We also performed a manual search for additional relevant studies using references of the included articles. The following search terms were used: (“pulse dose” or “high dose”), (“methylprednisolone” or “dexamethasone” or “hydrocortisone” or “prednisone” or “glucocorticoids” or “steroids”), and (“COVID” or “COVID‐19”). The search was not limited by language, study design, or country of origin. Supporting Information: Table S1 describes the full search terms used in each database searched.

### 2.2 Inclusion and exclusion criteria

All peer‐reviewed studies that compared PDS versus NPDS in COVID‐19 patients and reported one of the following outcomes: mortality, need for endotracheal intubation, length of stay (LOS), or adverse events were eligible for inclusion. PDS therapy should be clearly defined and must meet the following criteria: a dose of >250 mg of methylprednisolone (MTP) or 5–10 mg/kg/day of MTP or an equivalent dose of glucocorticoids (GC) must be administered to >75% of the PDS group for at least two consecutive days. NPDS cohort should receive any dose less than the PDS doses. We excluded single‐arm studies, case reports, case series, reviews, editorials, abstracts, and preprint studies.

### 2.3 Data extraction

The following data were extracted from the studies: first author name, publication year, country of origin, study design, sample size, gender of patients, mean age, and underlying comorbidities of the patients, including asthma and other chronic lung diseases, malignancy, coronary artery disease, and chronic kidney disease or acute kidney injury. We also obtained inclusion criteria in each study and respiratory support used in each study. For each arm of the study, the detailed PDS and NPDS treatment regimens were extracted. Outcomes measures were also retrieved, including mortality, need for endotracheal intubation, LOS, and superimposed infection.

### 2.4 Outcomes

The primary outcome of our study was mortality. The secondary outcomes were the need for endotracheal intubation, LOS, and adverse events in the form of superimposed infections or bacterial growth in cultured bodily fluids.

### 2.5 Statistical analysis

We performed a meta‐analysis of the included studies using Review Manager 5.3 (Cochrane Collaboration, Copenhagen, The Nordic Cochrane Centre) and Comprehensive Meta‐Analysis (Biostat). The random‐effects model was used to calculate the pooled risk ratio (RR) and mean difference (MD) with the corresponding confidence intervals (CIs) for proportional and continuous variables, respectively. A *p* < 0.05 was considered statistically significant. The heterogeneity of the effect size estimates across the studies was quantified using the Q statistic and *I* ^2^ (*p* < 0.10 was considered significant). A value of *I* ^2^ of 0%–25% indicates significant homogeneity, 26%–50% low homogeneity, and >50% indicates heterogeneity.^20^

### 2.6 Sensitivity and subgroup analyses

To confirm the robustness of the results, sensitivity analysis for all outcomes (mortality, need for endotracheal intubation, LOS, and adverse events) using a leave‐one‐out meta‐analysis was performed to see if it had a significant influence on the result of the meta‐analysis. Subgroup analysis was performed for studies that only included patients who received dexamethasone (DEXA) only in the NPDS group and based on the PDS therapy strategy (initial vs. rescue therapy) for mortality.

### 2.7 Bias assessment

We assessed the quality of the included studies using the Newcastle‐Ottawa Scale (NOS) for observational studies.^21^ Two authors (W. K. and A. B.) independently assessed each study for bias. Discrepancies were resolved by a third reviewer (O. S.). Publication bias was assessed for mortality qualitatively by visualizing the funnel plot and quantitively using Egger's regression analysis. A *p*‐value was generated using the Egger analysis, and a value of <0.05 was associated with significant publication bias.

## 3 RESULTS

### 3.1 Study selection

**Figure 1:**
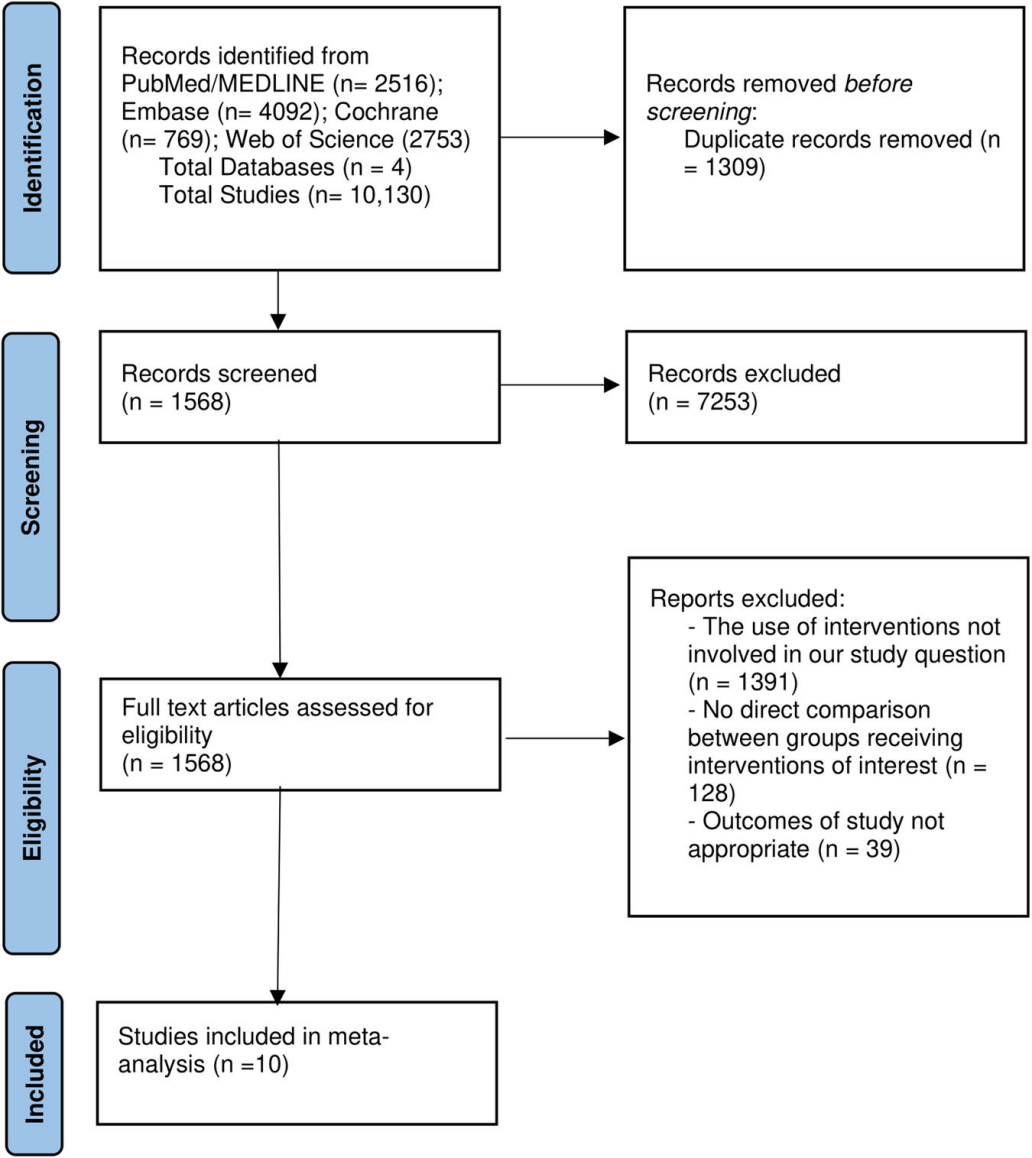
Preferred reporting items for systematic reviews and meta‐analyses flow diagram for the selection of studies.

A total of 10,130 studies were retrieved by our search strategy. Seven thousand two hundred fifty‐three studies were excluded based on the title and abstract review. A total of 1568 studies underwent full‐length review. Subsequently, we excluded 1558 studies because of the following: 1391 studies used inappropriate doses of steroids or lacked the appropriate comparison, 128 studies did not report data regarding the interventions of interest, and 39 studies were excluded due to lack of the appropriate outcomes of interest. Eventually, 10 studies met our inclusion criteria and were included in the meta‐analysis.^14^, ^15^, ^16^, ^17^, ^18^, ^19^, ^22^, ^23^, ^24^, ^25^ Figure 1 shows the preferred reporting items for systematic reviews and meta‐analyses (PRISMA) flow chart that illustrates how the final studies were selected.

### 3.2 Study and patient characteristics

Table 1 shows the baseline characteristics of the studies included in the meta‐analysis. All the studies were published between August 2020 and December 2021 and included COVID‐19 patients confirmed by laboratory testing or imaging. Based on country of origin: five studies originated from Europe (Italy: 1; Spain: 2; and Turkey: 2), one from Morocco, one from Colombia, one from Japan, one from Pakistan, and one from the United States. Nine studies were retrospective cohort, while one was an ambispective cohort.

**Table 1:** Study and patient characteristics of the included studies.

Study (year)	Batirel (2021)	Fernandez‐Cruz (2020)	El mezzeoui (2021)	Gundogdu (2021)	Jamil (2021)	Monreal (2021)	Pinzon (2021)	Toda (2021)	Umbrello (2021)	Yaqoob (2021)
Study design	Retrospective cohort	Retrospective cohort	Retrospective cohort	Retrospective cohort	Retrospective cohort	Retrospective cohort	Ambispective cohort	Retrospective cohort	Retrospective cohort	Retrospective cohort
Country	Turkey	Spain	Morocco	Turkey	Pakistan	Spain	Colombia	Japan	Italy	USA
Total patient (PDS/NPDS)	300 (150/150)	396 (86/310)	513 (283/230)	400 (200/200)	433 (209/224)	573 (177/396)	216 (105/111)	17 (8/9)	80 (22/58)	136 (49/87)
PDS group age, mean ±** **SD, years	59.4 ± 17.0	65.4 ± 12.9 for all steroid recipients	62.9 ± 15.7	66.9 ± 12.2	55.9 ± 17.0 for all steroid recipients	65.9 ± 15.7	64.0 ± 6.0	59.5 ± 17.8	61 ± 8	66 ± 13
NPDS group age, mean ±** **SD, years	59.9 ± 16.6		64.4 ± 14.9	71.3 ± 11.1		63.3 ± 13.4	63.4 ± 8.3	77.0 ± 14.0	60 ± 10	60 ± 16
Male in PDS group, *n* (%)	100 (67)	276 (69.7)	190 (67.3)	135 (67.5)	100 (23.1) for all steroid recipients	150 (84.8)	67 (63.8)	8 (100)	19 (86.4)	27 (55)
Male in NPDS group, *n* (%)	100 (67)		162 (70.4)	111 (55.5)		278 (70.2)	60 (54.1)	6 (67)	48 (84.8)	60 (69)
Patient inclusion criteria	Nonmechanically ventilated patients with no current bacterial or fungal infections, and not having received any other anti‐cytokine, anti‐inflammatory or a steroid regimen other than the intended group defined steroid treatments	Patients that had evidence of ARDS and/or had laboratory evidence of hyper‐inflammatory syndrome	All patients initially admitted to the ICU and stayed in the hospital for >7 days	Patients who did not receive another anti‐inflammatory, anti‐cytokine drug, or receive any inappropriate doses of glucocorticoids that were intended as part of the study	Patients with evidence of ARDS or >50% of total lung fields showing infiltrates on chest x‐ray. Patients also had to not have received previous recent course of steroids, tocilizumab, or have any contraindications to steroids treatment	Patients who did not receive remdesivir and had confirmed ARDS via the Berlin criteria	Patients who required supplemental oxygen, were able to receive adequate steroid treatment as defined by the treatment protocol of the study	NR	Mechanically ventilated and deeply sedated patients with moderate to severe ARDS	Patients admitted to the ICU with a primary diagnosis of COVID‐19 pneumonia to the ICU, were started on steroids within 48 h of ICU admission and received > 1 pulse dose of steroid
Hospital location	Non‐ICU patients	All except 30 patients were non‐ICU	ICU patients	ICU patients	ICU patients	NR	Non‐ICU patients	NR	ICU patients	ICU patients
Ventilation status before steroid regimen	No patient on IMV	Most patients not on IMV	NPDS group: No patient on IMV	NR	12/433 patients on IMV; 32/433 patients on NIV	NPDS group: 11 patients on NIV or IMV	NR	No patient on IMV	All patients were on IMV	NPDS group: 70 patients on IMV
						PDS group: 7 patients on NIV or IMV			
										PDS group: 45 patients on IMV
			PDS group: Three patients on IMV							
Days from symptom onset to intervention (PDS/NPDS)	6.5 ± 4.9/7.0 ± 4.5	10.4 ± 3.7 for all steroid recipients	NR	NR	NR	8.4 ± 3.7/8.7 ± 4.5	NR	7.5 ± 6.8/7.0 ± 3.0	NR	NR
Other relevant treatments specified	Antibiotics (not specified), remdesivir, HCQ, favipiravir, lopinavir‐ritonavir	Azithromycin, doxycycline, tocilizumab, anakinra, HCQ, lopinavir‐ritonavir	NR	Anakinra, tocilizumab, favipiravir	NR	Antibiotics (not specified), remdesivir, HCQ, lopinavir‐ritonavir	Antibiotics (not specified)	Remdesivir, favipiravir	Tocilizumab	Azithromycin, HCQ
PDS regimen	Patients who did not respond positively to NPDS therapy after 3 days of treatment with either clinical or radiologic improvement were given rescue PDS therapy with 250 mg/day of MTP for 3 days, followed again by NPDS therapy to complete a 10‐day course of steroid treatment	Patients received 3 days of PDS therapy with either 250 mg/day (62.5%) of MTP or 500 mg/day (17.1%) of MTP	6 mg/kg/day of DXM for 7 days	1 g/day of MTP for 3 days followed by 80 mg/day of MTP	500 mg/day of MTP for 10 days	250 mg to 1 g per day of MTP or an equivalent steroid dose for 1 or more consecutive days	IV 250 mg to 500 mg per day of MTP for 3 days. Followed by oral prednisone 50 mg/day for the next 14 days	Patients who did not respond positively to NPDS therapy and went on to require O_2_ > 8 L/min and needed HFNO, or MV were given rescue PDS therapy with either 500 mg/day of MTP for those <75 kg or 1 g/day of MTP for those >75 kg. Followed by tapering steroid treatment with DXM based on clinical condition	Patients who did not respond positively to NPDS treatment and developed worsening ARDS were given 1 g/day of IV MTP for 3 days, within the first 10 days of initiating high dose steroid therapy, followed by resumption of NPDS therapy to complete a 14‐day course	Patients received 1 g/day of MTP. Timing and number of doses varied based on clinical condition
						Days of mean ±** **SD for treatment was 5.8 ± 9.0				
		20.1% of patients received 3 days of <250 mg/day of MTP								
NPDS regimen	DXM 6 mg/day or equivalent dose of any steroid for 10 days	1 mg/kg/day of MTP or an equivalent steroid	1 mg/kg/day of MTP for 7 days	DXM 8 mg/day or MTP 80 mg/day	DXM 6 mg or more per day for 10 days	0.5–1.5 mg/kg/day of MTP or an equivalent steroid dose	IV DXM 6 mg/day for 10 days	DXM 6 mg/day until clinical improvement	DXM 20 mg/day for the first 7 days, followed by 10 mg/day for the next 7 days to complete a 14‐day treatment course. Or MTP 1 mg/kg/day for 14 days	0.5–2 mg/kg/day of MTP or an equivalent steroid dose
					176 patients received DXM 6 mg/day					
		22.5% of NPDS patients went on to receive rescue PDS therapy								
					48 patients received >6 mg/day DXM					
Follow‐up duration	28 days	30 days	NR	NR	30 days	Median 21 days	30 days	Mean 27 days (range: 14–46)	NPDS group: Median 27 days	28 days
									PDS group: Median 21 days	
*Underlying comorbidities*										
Asthma and other chronic lung diseases (PDS/NPDS)	15/21	71 of the total 396 steroid recipients	NR	NR	38 of the total 433 steroid recipients	32/62	11/12	2/1	NR	1/11
Malignancy (PDS/NPDS)	14/16	49 of the total 396 steroid recipients	NR	NR	18 of the total 433 steroid recipients	22/35	6/7	2/1	NR	9/6
CAD (PDS/NPDS)	27/30	72 of the total 396 steroid recipients	39/32	NR	42 of the total 433 steroid recipients	36/63	9/10	NR	NR	2/10
CKD or AKI (PDS/NPDS)	10/8	24 of the total 396 steroid recipients	17/15	NR	56 of the total 433 steroid recipients	17/28	7/13	3/2	NR	2/12

Abbreviations: AKI, acute kidney injury; ARDS, acute respiratory distress syndrome; CAD, coronary artery disease; CKD, chronic kidney disease; COVID‐19, coronavirus disease 2019; DXM, dexamethasone; HCQ, hydroxychloroquine; ICU, intensive care unit; IMV, invasive mechanical ventilation; IV, intravenous; MTP, methylprednisolone; NIV, noninvasive ventilation; NPDS, nonpulse dose steroids; NR, not reported; PDS, pulse dose steroids; SARS‐CoV‐2, severe acute respiratory syndrome coronavirus 2; SD, standard deviation.

A total of 3065 patients (1289 patients received PDS and 1776 received NPDS) were included, with males representing 61.8% of the total patients. The mean age of the patients in the PDS group was 56.1 years, and 65 years in the NPDS group. The follow‐up period across the studies ranged from 14 to 46 days.

### 3.3 Mortality

**Figure 2:**
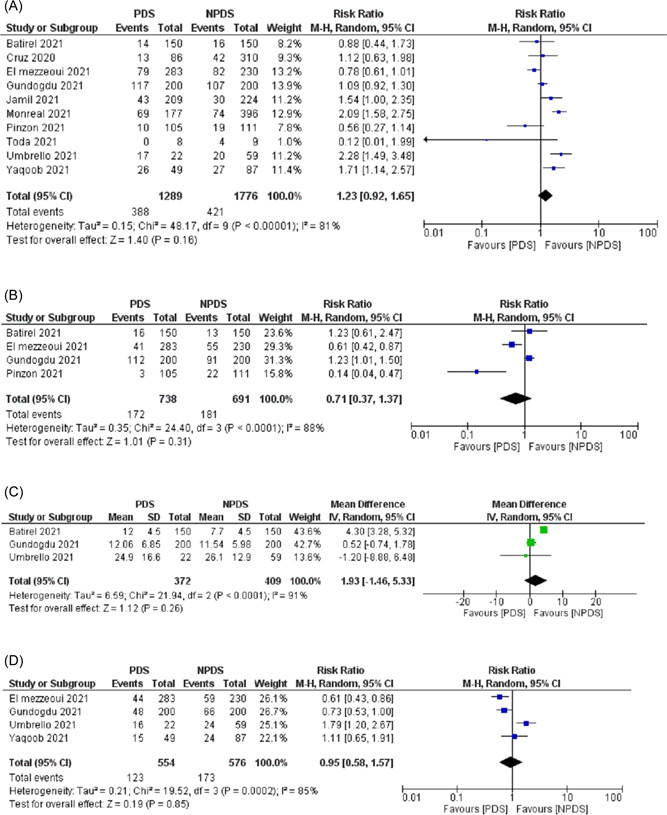
Forest plot comparing PDS and NPDS regarding (A) mortality, (B) need for endotracheal intubation, (C) length of hospital stay, and (D) adverse events. NPDS, nonpulse dose steroids; PDS, pulse dose steroids.

**Figure 3:**
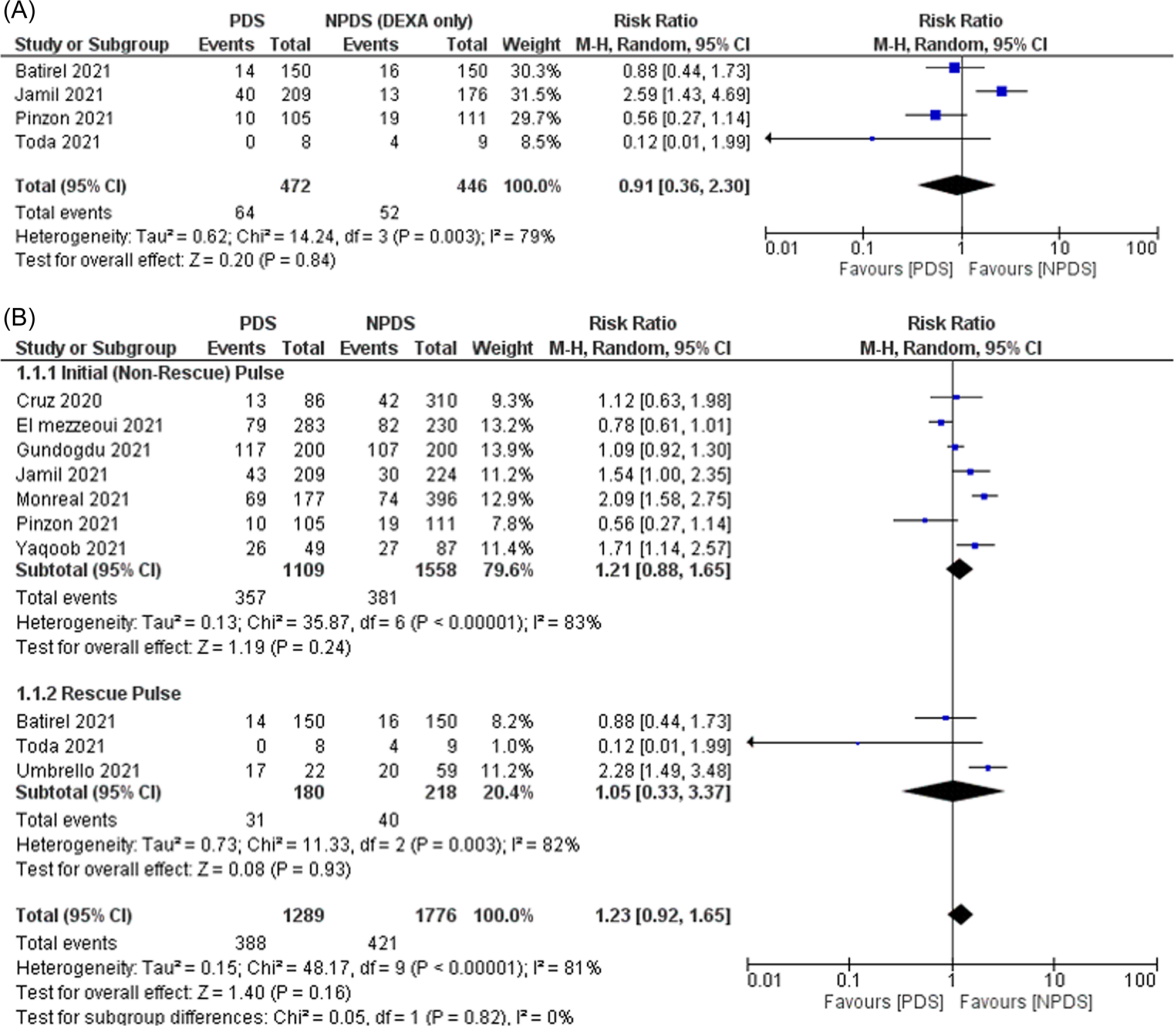
(A) Subgroup analysis of studies that included only dexamethasone in the NPDS group for mortality, (B) Subgroup analysis based on the strategy of pulse‐dose steroid therapy (initial vs. rescue therapy) for mortality. NPDS, nonpulse dose steroids; PDS, pulse dose steroids.

Table 2 summarizes the outcomes of the individual studies included in the meta‐analysis. All 10 studies^14^, ^15^, ^16^, ^17^, ^18^, ^19^, ^22^, ^23^, ^24^, ^25^ reported the mortality rate. The mortality rate was 30.1% in the PDS group compared to 23.7% in the NPDS group. There was no significant difference in the mortality rate between the two groups (RR: 1.23, 95% CI: 0.92–1.65, *p* = 0.16, *I* ^2^ = 81%, Figure 2A). A subgroup analysis of studies that included only DEXA in the NPDS group showed similar mortality rates between the two groups (RR: 0.91, 95% CI: 0.91–2.30, *p*  = 0.84, *I* ^2^  =  79%, Figure 3A). Subgroup analysis also showed no significant difference if PDS was given as initial therapy or rescue therapy (Figure 3B). A leave‐one‐out sensitivity analysis showed consistent results (Supporting Information: Figure S1A).

**Table 2:** Primary and secondary outcomes of the included studies.

Study (year)	In‐hospital mortality		Need for mechanical ventilation		Hospital length of stay (days)		Adverse events	
	PDS (%)	NPDS (%)	PDS (%)	NPDS (%)	PDS (SD)	NPDS (SD)	PDS (%)	NPDS (%)
Batirel (2021)	14/150 (9.3)	16/150 (10.7)	16/150 (10.7)	13/150 (8.7)	12 (4.5)	7.7 (4.5)	‐	‐
Cruz (2020)	13/86 (15.1)	42/310 (13.5)	‐	‐	‐	‐	‐	‐
El mezzeoui (2021)	79/283 (27.9)	82/230 (35.7)	41/283 (14.5)	55/230 (23.9)	‐	‐	44/283 (15.5)	59/230 (25.7)
Gundogdu (2021)	117/200 (58.5)	107/200 (53.5)	112/200 (56.0)	91/200 (45.5)	12.06 (6.85)	11.54 (5.98)	48/200 (24)	66/200 (33)
Jamil (2021)	43/209 (20.6)	13/176 for the DXM 6 mg/day group. 17/48 for those receiving >6 mg/day DXM. Overall, 30/224 (13.4)	‐	‐	‐	‐	‐	‐
Monreal (2021)	69/177 (39.0)	74/396 (18.7)	‐	‐	‐	‐	‐	‐
Pinzon (2021)	10/105 (9.5)	19/111 (17.1)	3/105 (2.9)	22/111 (19.8)	‐	‐	‐	‐
Toda (2021)	0/8 (0.0)	4/9 (44.4)	‐	‐	‐	‐	‐	‐
Umbrello (2021)	17/22 (77.3)	20/59 (33.9)	‐	‐	24.9 (16.6)	26.1 (12.9)	16/22 (72.7)	24/59 (40.7)
Yaqoob (2021)	26/49 (53.1)	27/87 (31.0)	‐	‐	‐	‐	15/49 (30.6)	24/87 (27.6)

Abbreviations: NPDS, nonpulse dose steroids; PDS, pulse dose steroids.

### 3.4 Need for endotracheal intubation

Across the four studies^14^, ^15^, ^17^, ^19^ that reported the intubation rate; 23.3% of patients who received PDS required intubation compared to 26.2% in patients who received NPDS. The need for endotracheal intubation was similar between PDS and NPDS groups (RR: 0.71, 95% CI: 0.37–1.137, *p* = 0.31, *I* ^2^ = 88%, Figure 2B). Consistent results were shown on sensitivity analysis (Supporting Information: Figure S1B).

### 3.5 Length of hospital stay

Three studies^17^, ^19^, ^23^ reported the LOS. There was no significant difference in the LOS between the two groups (MD: 1.93 days; 95% CI: −1.46–5.33; *p* = 0.26, *I* ^2^ = 91%, Figure 2C). The results were consistent on the leave‐one‐out sensitivity analysis (Supporting Information: Figure S1C).

### 3.6 Adverse events

Four studies^14^, ^19^, ^23^, ^24^ reported the adverse events in the form of superimposed infection or bacterial growth in bodily fluid cultures. These events were similar between the PDS and NPDS groups (RR: 0.93, 95% CI: 0.56–1.57, *p* = 0.80, *I* ^2^ = 86%, Figure 2D). However, on a leave‐one‐out sensitivity analysis, removal of Umbrello et al.^23^ moved the overall effect to favor PDS with an RR of 0.74 (95% CI: 0.56–0.99, *p* = 0.05, *I* ^2^ = 41%), suggesting that Umbrello et al. was partly the reason for the significant between‐study heterogeneity (Supporting Information: Figure S1D).

### 3.7 Quality and publication bias assessment

We followed the PRISMA statement flow diagram model to select the final studies. Two investigators (W. K. and A. B.) independently performed the search and shortlisted the studies for final review. Discrepancies were resolved by a third reviewer (O. S.). Quality assessment scoring of the included studies was performed using the NOS for assessing nonrandomized studies, and the scores are summarized in Supporting Information: Table S2. All the included studies^14^, ^15^, ^16^, ^17^, ^18^, ^19^, ^22^, ^23^, ^24^, ^25^ were of high quality. There was a visible asymmetry in the funnel plot of the studies that reported mortality, which may suggest the presence of publication bias (Supporting Information: Figure S2). However, Egger's regression analysis did not demonstrate statistically significant publication bias (*p* = 0.86).

## 4 DISCUSSION

This is the first meta‐analysis comparing PDS and NPDS regimens in patients with COVID‐19 and showed no significant differences between PDS and NPDS regimens in terms of mortality, need for endotracheal intubation, LOS, and adverse events.

Since the RECOVERY trial showed a mortality benefit with steroid therapy due to its anti‐inflammatory and immunomodulatory effects,^6^ steroid therapy (equivalent dose of 6 mg dexamethasone [DEXA]) became a standard treatment in most hospitals for COVID‐19 patients requiring oxygen support. A recent meta‐analysis by Li et al.^7^ revealed consistent results with a significant reduction in mortality among patients with severe COVID‐19, especially when administered earlier.

Although steroid therapy is a common practice in the current management of COVID‐19, there are current debates on the dose of the corticosteroids that should be used in these patients. In an RCT by Edalatifard et al.,^26^ including 68 patients with COVID‐19, the PDS group (received methylprednisolone therapy at a dose of 250 mg/day for 3 days) had a lower mortality rate and shorter recovery duration as compared to the control group. The rationale of using PDS therapy is to get a quicker and stronger anti‐inflammatory response, thus reducing the need for a prolonged course of steroids.^9^

Fernandez‐Cruz et al.^18^ published the first comparative study between PDS and NPDS regimens in 2020, demonstrating no difference in mortality between PDS and NPDS regimens (15.1% and 13.5%, respectively). Since then, there have been a number of studies comparing the two regimens with controversial results. While several studies showed similar mortality rates between PDS and NPDS regimens,^15^, ^16^, ^17^, ^18^, ^19^ El mezzeoui et al. demonstrated significantly decreased mortality in the PDS group compared to NPDS. On the contrary, Umbrello et al. found that PDS was associated with increased mortality compared to NPDS. Given the contradicting results of the studies in the literature, we conducted this meta‐analysis to provide the first comprehensive evaluation and comparison of the two steroid therapy regimens for COVID‐19 to address critical knowledge gaps in the management of COVID‐19.

Our study results were consistent with a cohort study conducted by Ho et al. in 2003,^10^ which showed no differences in mortality or need for endotracheal intubation between PDS and NPDS in the management of patients with SARS. In this meta‐analysis, we found similar results to those from a study by Gundogdu et al.,^19^ which showed that PDS and NPDS were associated with similar mortality (58.5% and 53.5%, respectively). Furthermore, our meta‐analysis results remained consistent on subgroup analysis based on the strategy of PDS therapy (initial vs. rescue therapy). Comparable to our results, Gundogdu et al.^19^ demonstrated similar rates of need for endotracheal intubation and LOS between the PDS and NPDS regimens.

There are several adverse events to using steroids, such as superimposed infections/bacterial overgrowth, hyperglycemia, and myopathy. In contrary to Umbrello et al.,^23^ which showed an increased risk of infections with PDS regimen in addition to mortality, our meta‐analysis showed similar rates of infection between the two regimens. The increased rates of infections within the PDS group in that study could be attributed to the fact that PDS was used as a rescue therapy after lack of response to NPDS regimen, indicating that the PDS group patients were sicker and had more severe features of COVID‐19.^23^ In addition, PDS group had higher levels of C‐reactive protein, IL‐6, and d‐dimer and a lower Partial pressure of arterial oxygen (PaO_2_).^23^ Interestingly, we found that the infection rate was significantly lower in the PDS group upon removal of that study. This might be explained by the short duration of the PDS regimen.

However, serious side effects such as sudden death, cardiac arrhythmias, and seizures could occur with the PDS regimen, especially when the single dose of methylprednisolone exceeds 500 mg/day.^27^ Thus, we believe that there is an urgent need for clear protocols to guide the practice of steroid therapy in COVID‐19 patients, especially the optimal dosage and duration of steroid therapy. Future RCTs are needed to determine the efficacy and safety of PDS versus NPDS regimens in the management of COVID‐19.

Several limitations of this study should be acknowledged. First, because the literature lacks RCTs, our meta‐analysis included only observational studies. Future RCTs are warranted to confirm our findings. Second, even though the random‐effects model was used in our analysis, there was significant heterogeneity noted in all the outcomes. This might be driven by differences in patient characteristics, inconsistent follow‐up duration, the difference in the location of the patients (hospital ward vs. ICU), use of other drugs for COVID‐19 treatment, and lack of standardized dosages and type of the steroids used in the included studies. Third, we could not evaluate the side effects of PDS versus NPDS other than superimposed bacterial infection, such as hyperglycemia or myopathy due to limited reported data. Lastly, the lack of patient‐level data did not allow to control for possible variations in baseline characteristics or optimal dosage of steroids and duration of treatment because other medications used for COVID‐19 might have been different and not standardized between patients, which might introduce potential bias.

Despite the limitations, our study has significant strengths. First, we included a total of 10 studies with over 3000 patients with COVID‐19. To our knowledge, this is the first meta‐analysis comparing the effect of PDS versus NPDS on clinical outcomes in COVID‐19 patients. Although heterogeneity was noted, we performed sensitivity analyses for the primary and secondary outcomes and subgroup analysis for mortality to evaluate the robustness of our results. Consistent results were observed on sensitivity analysis for all the outcomes and subgroup analysis of studies that included only DEXA in NPDS group and based on the strategy of PDS therapy (Initial vs. rescue therapy) for mortality. Lastly, all the studies in our meta‐analysis were of high quality based on the NOS quality assessment.

In conclusion, our meta‐analysis demonstrates no significant differences in mortality, need for mechanical ventilation, LOS, and adverse events between PDS and NPDS regimens in the management of COVID‐19. However, given the observational nature of the studies, RCTs are warranted to validate our findings.

## AUTHOR CONTRIBUTIONS

Waleed Khokher and Azizullah Beran conceived and designed the study and critically revised the manuscript. Waleed Khokher and Azizullah Beran designed the study, collected, analyzed, interpreted the data, and drafted the manuscript. Saffa Iftikhar, Omar Srour, Mohammed Mhanna, Saif‐Eddin Malhas, Sapan Bhuta, Nithin Kesireddy, Dipen Patel, Elizabeth Borchers, and Cameron Burmeister collected the data and reviewed the literature. Fadi Safi and Ragheb Assaly supervised and critically revised the manuscript. All authors had access to the data and a role in writing the manuscript. All authors read and approved the final manuscript.

## CONFLICTS OF INTEREST

The authors declare no conflicts of interest.

## ETHICS STATEMENT

This study was deemed exempt by the Institutional Review Board of the University of Toledo, as it was a meta‐analysis of published studies that included deidentified patient information.

## Supporting information

Supporting information.

## Data Availability

All data were available to all authors of the manuscript.
