# Supplementary material for: Pulse versus nonpulse steroid regimens in patients with coronavirus disease 2019: A systematic review and meta‐analysis
Source: J Med Virol. 2022 May 9;94(9):4125–37. doi: 10.1002/jmv.27824 (PMC9347719; doi:10.1002/jmv.27824)
Supplement: Supplementary file 1 — Supporting information. [file JMV-94-4125-s001.docx]

# **Supplementary Appendix**

# **Pulse versus Non-pulse Steroid Regimens in Patients with Coronavirus Disease 2019: A Systematic Review and Meta-Analysis**

Waleed Khokher MD, Azizullah Beran MD, Saffa Iftikhar MD, Saif-Eddin Malhas MD, Omar Srour MD, Mohammed Mhanna MD, Sapan Bhuta MD, Dipen Patel MD, Nithin Kesireddy MD, Cameron Burmeister MD, Elizabeth Borchers MS3, Ragheb Assaly MD, Fadi Safi MD

**Supplementary tables**

| Title | Page |
| --- | --- |
| Supplementary table 1: Search strategy used in each database searched. | 2 |
| Supplementary table 2: The Newcastle-Ottawa Scale (NOS) for assessing the quality of observational studies. | 3 |

**Supplementary figures:**

| Title | Page |
| --- | --- |
| Supplementary figure 1: Leave-one-out sensitivity analysis for: A) mortality, B) need for endotracheal intubation, C) length of hospital stay, and D) adverse events. | 4 |
| Supplementary figure 2: Funnel plot showing publication bias analysis for mortality. | 5 |

Supplementary table 1: Search strategy used in each database searched.

| Database | Search Strategy | Articles retrieved |
| --- | --- | --- |
| PubMed/MEDLINE | ("glucocorticoids"[All Fields] OR "steroids"[All Fields] OR "hydrocortisone"[All Fields] OR "methylprednisolone"[All Fields] OR "dexamethasone"[All Fields]) AND ("pulse dose"[All Fields] OR "high dose"[All Fields] OR "pulse"[All Fields] OR "short term”[All Fields] OR "long term"[All Fields]) AND ("COVID"[All Fields] OR "COVID-19 pneumonia"[All Fields] OR "SARS-CoV"[All Fields] OR "Viral COVID pneumonia"[All Fields] OR "Viral Pneumonia"[All Fields]) AND ("clinical deterioration"[All Fields] OR "hospitalization"[All Fields] OR "shortness of breath"[All Fields] OR "supplemental oxygen"[All Fields] OR "hospital stay"[All Fields] OR "intensive care unit"[All Fields] OR "ICU admission"[All Fields]) AND ("intubated"[All Fields] OR "non-intubated” [All Fields] OR "mechanically ventilated"[All Fields] OR "ventilated"[All Fields]) AND ("ARDS"[All Fields] OR "acute respiratory distress syndrome"[All Fields] OR “respiratory distress syndrome"[All Fields]) AND ("mortality” [All Fields] OR "death"[All Fields] OR “ICU mortality"[All Fields] OR "hospital mortality”[All Fields]) | 2516 |
| Embase | (‘glucocorticoids OR ‘methylprednisolone’ OR ‘hydrocortisone’ OR ‘dexamethasone’ OR ‘steroids’) AND ('coronavirus' OR 'COVID-19' OR 'SARS-CoV 2’) AND (‘pulse dose’ OR 'high dose’) AND ('intensive care unit' OR 'intensive care’ OR ‘mechanical ventilation' OR “intubated’ OR ‘mortality OR ‘death’) | 4092 |
| Web of Science | (‘glucocorticoids OR ‘methylprednisolone’ OR ‘hydrocortisone’ OR ‘dexamethasone’ OR ‘steroids’) AND ('coronavirus' OR 'COVID-19' OR 'COVID’ OR ‘SARS-CoV 2’) AND (‘pulse dose’ OR 'high dose’) | 2753 |
| Cochrane CENTRAL | "Mortality" in Title Abstract Keyword AND "Coronavirus" in Title Abstract Keyword AND “steroids” in Title Abstract Keyword AND “high dose” in Title Abstract Keyword | 769 |

Supplementary table 2: The Newcastle-Ottawa Scale (NOS) for assessing the quality of observational studies.

|  | Selection | | | | | Outcome | | | |
| --- | --- | --- | --- | --- | --- | --- | --- | --- | --- |
| Study, Year | Representativeness of the exposed cohort | Selection of the non-exposed cohort | Ascertainment of exposure | Outcome not present at baseline | Comparability of the cohort | Assessment of outcome | Enough follow up duration | Adequate follow-up | Total score |
| Batirel 2021 | * | * | * | * | * | * | * | * | 8 |
| Cruz 2020 | * | * | * | * | * | * | * | * | 8 |
| El mezzeoui 2021 | * | * | * | * | * | * | * | * | 8 |
| Gundogdu 2021 | * | * | * | * | 0 | * | * | * | 7 |
| Jamil 2021 | * | * | * | * | * | * | * | * | 8 |
| Monreal 2021 | * | * | * | * | * | * | * | * | 8 |
| Pinzon 2021 | * | * | * | * | * | * | * | * | 8 |
| Toda 2021 | * | * | * | * | 0 | * | * | * | 7 |
| Umbrello 2021 | * | * | * | * | 0 | * | * | * | 7 |
| Yaqoob 2021 | * | * | * | * | * | * | * | * | 8 |

Each asterisk represents one star in the Newcastle-Ottawa Scaling System (NOS). The maximum stars are 2 for comparability and 1 are for all other categories. Each star counts towards the total score. Score of 5 to 6 considered as moderate quality and 7 to 9 as high quality.


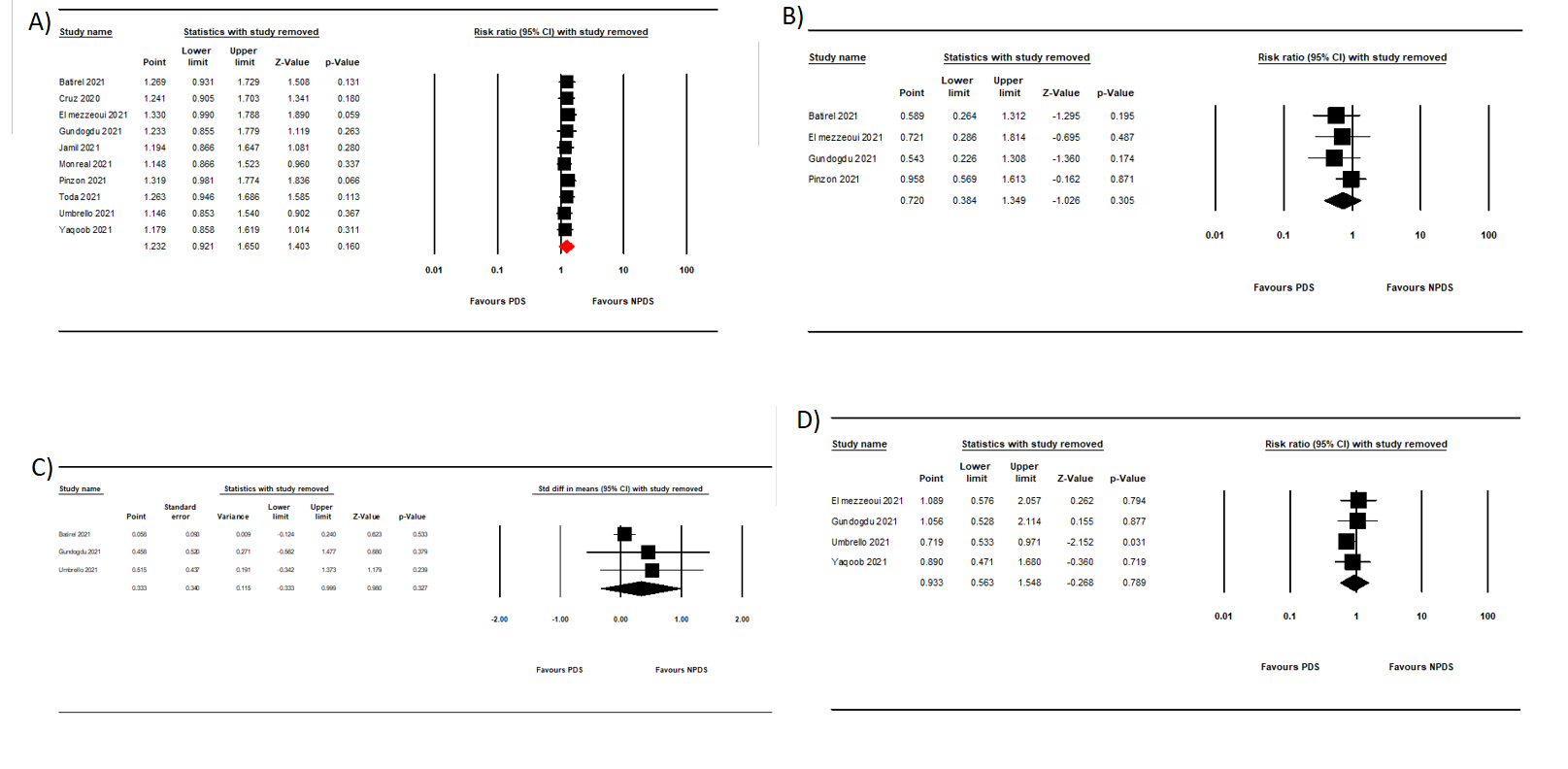
Supplementary figure 1: Leave-one-out sensitivity analysis for: A) mortality, B) need for endotracheal intubation, C) length of hospital stay, and D) adverse events.

 Supplementary figure 2: Funnel plot showing publication bias analysis for mortality.
